# Macrophages unlock progression of breast cancer cells experiencing *matrigel-segregation* in transplantation models

**DOI:** 10.1038/s41598-017-11403-w

**Published:** 2017-09-08

**Authors:** Misa Ogura, Victoria L. Bridgeman, Ilaria Malanchi

**Affiliations:** Tumour Host Interaction Lab, The Francis Crick Institute, 1 Midland Rd, NW1 1AT London, UK

## Abstract

Basement membrane matrix proteins, such as matrigel, are able to improve the efficiency of tumour transplantation. This assay represents the gold standard to measure tumour initiation potential *in vivo* of a limited number of cancer cells. However, in culture conditions, matrigel directly signals to cancer cells altering their phenotype. We here investigate how matrigel influences the tumour reconstitution dynamics of breast cancer cells *in vivo*. This is particularly relevant in the setting of limiting dilution assay where cells are transplanted in a relatively high amount of Matrigel. We show that matrigel initially induces a normalized growth of transplanted MMTV-PyMT breast tumours cells. This occurs in the context of a *matrigel-segregation effect* where cancer cells are transiently isolated from host tissue. We identify macrophages as gatekeepers of the cancer-host cell interaction: depriving transplants from macrophages locked cancer cells in this isolated environment where they fail to form tumours despite retaining their intrinsic tumorigenic potential. This is a decisive proof of concept that cancer cells’ malignant behaviour can be dominated by their microenvironment. Moreover, considering that diverse breast cancer cells are differently subjected to a *segregation effect*, this needs to be considered when comparing tumour initiation potential of different cancer cells.

## Introduction

Tumour is a highly complex structure, where a heterogeneous assembly of host tissue maintains cancer cell growth and fosters different characteristics and cancer cell behaviours. This intricate microenvironment, composed of a plethora of different cell types embedded in a remodelled extracellular matrix (ECM) protein environment, is involved in every aspect of cancer growth and progression^1^. Cancer growth initiation requires a concomitant modification of its surrounding and the establishment of a favourable crosstalk with host tissue cells is prerequisite to initiate secondary growth^2^. Importantly, as a consequence of this persistent crosstalk, cancer cells and their surrounding microenvironment dynamically co-evolve over time allowing tumour progression^3^.

Most importantly, whereas these broad tissue microenvironment modifications and abnormal stromal components are vital for carcinogenesis, a normal stromal context acts to suppress tumour development^4^. The crosstalk between different tissue cells is important also for the maintenance of a normal tissue organization. Mammary gland tissue comprises a ductal and lobular structure where cells are organized in two layers: the inner layer of cells called luminal epithelial cells (K8 positive) are responsible for the milk production while the outer layer of cells, called myoepithelial cells (K5 and K14 positive) have the potential to contract and favour the flux of the milk in the mammary ducts. Myoepithelial cells also secrete many proteases allowing the remodelling of the mammary gland and the growth of new ducts or terminal lobules. Interestingly, myoepithelial cells have been reported to express proteins suppressing the tumorigenic potential of tumour initiating cells^5–7^. This suggests that the normal tissue infrastructure maintains a normal phenotype and buffers the early consequence of genetic mutations. Indeed, epithelial cells harbouring oncogenic genetic abnormalities can be found in normal skin^8^.

Tumour cells orchestrate their own surrounding where all cells respond and function to support growth and progression. ECM is an active component of the tumour microenvironment and both cancer and host derived cells continuously shape its composition. The communication between the cancer cells and their microenvironment is essential not only for their growth and progression, but also for the maintenance of the already achieved aggressive behaviour. Studies from the Bissel’s group have shown how the integration of adhesion signalling mediated by ECM proteins can rewire cancer cells phenotype and resistance to apoptosis *in vitro* ^9, 10^. This and similar studies also advocate for the idea of a dominant “normalizing” microenvironment over the malignant behaviour of cancer cells^4^. Nevertheless, to date fully developed malignant cancer cells from an aggressive carcinoma have never been “rewired” by a “normalizing” microenvironment *in vivo*.

In line with the evidence that ECM components are able to change the gene expression and influence tumour behaviour^11^, cells within the tumour microenvironment rearrange the ECM composition to support cancer growth and progression^12, 13^. Interestingly, the use of basement membrane matrix proteins, such as matrigel, was proven to increase growth initiation of tumour cells in recipient mice^14^. Therefore, especially for breast cancer, matrigel is now commonly used for tumour transplantations, which is also the gold standard assay to test tumour initiation potential of low number of cancer cells *in vivo* ^2, 15, 16^. However, whether matrigel influences the dynamics of mammary tumour reconstitution upon transplantation was never investigated.

By using a spontaneous genetic mouse model of breast cancer, we now show that primary carcinoma cells from malignant cancers transplanted in matrigel, are initially “rewired” and adopt a normalized ductal-like growth *in vivo*. This occurs in the context of a *matrigel-segregation effect* where cancer cells are transiently isolated from the host environment. This effect is overcome by host cells infiltration, which leads to the reconstitution of tumour associated stromal compartment and cancer growth.

Macrophages are a dominant and critical innate immune-component in the tumour microenvironment^17, 18^. They have been reported to be fundamental for tumour progression and growth as well as for supporting the resistance to anticancer therapies^18–20^. Their role in human cancer progression was also described using transplantation models^21^. Here we report that macrophages are the most abundant cells infiltrating matrigel plugs used to transplant breast carcinoma cells and that they are essential to trigger the reconstitution of the complex tumour microenvironment allowing aggressive tumour re-establishment. When deprived of macrophage infiltration, breast cancer cells that are heavily subjected to “normalizing” signals of basement membrane proteins, remain subjected of the *matrigel-segregation effect* and conditionally unable to exploit their intrinsic tumorigenic potential.

In addition to highlighting the fundamental role of macrophages in the tumour growth, our study represents a decisive proof of concept of the dominant impact of the tumour microenvironment not only in tumour progression, but also in the persistence of cancer cells malignant behaviour.

## Results

### Cancer cells derived from metastatic tumours recapitulate the spontaneous multistep process when transplanted in matrigel plug

**Figure 1:**
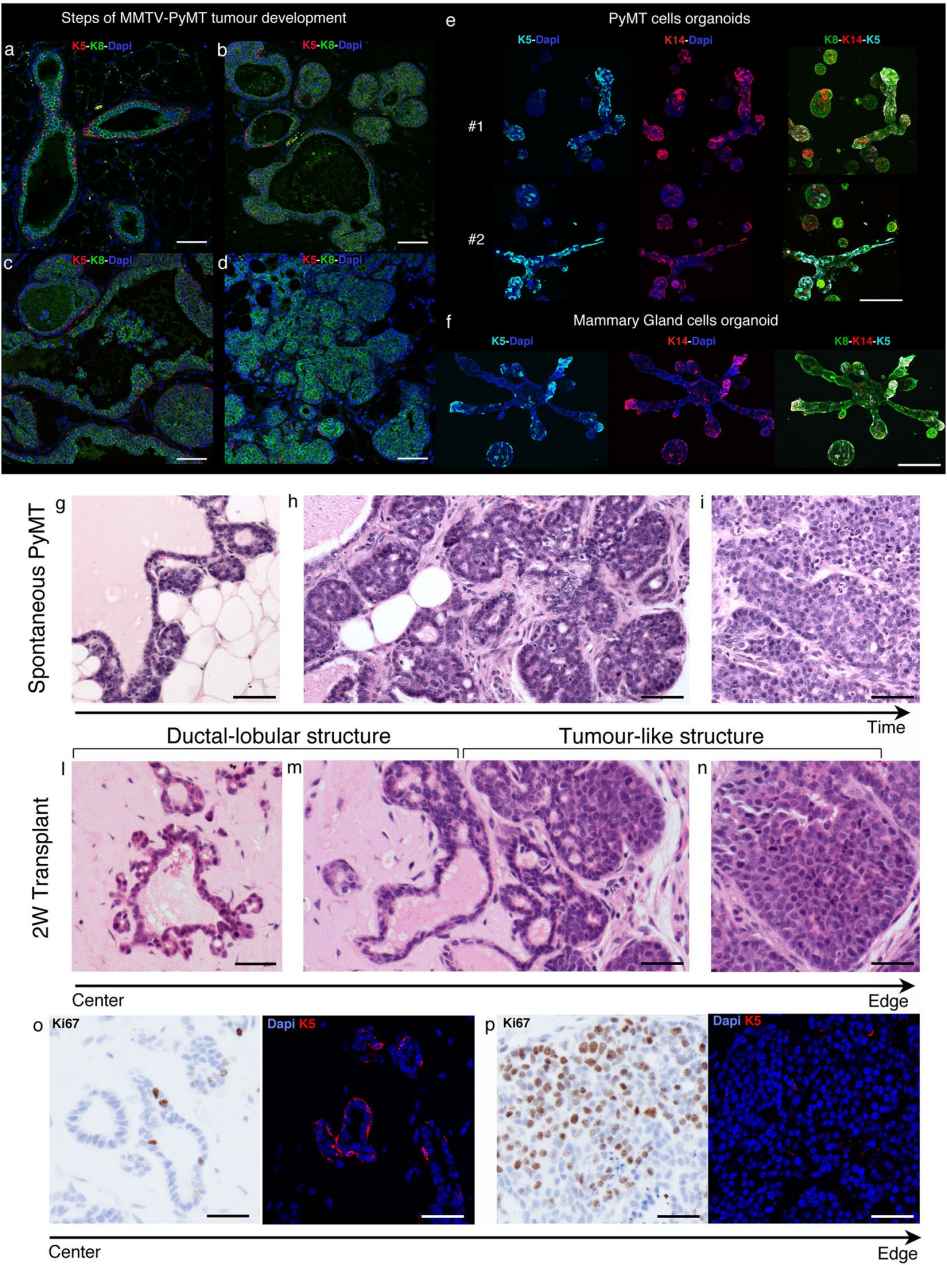
Development of spontaneous and transplanted MMTV-PyMT tumours. (**a**–**d**) Immunofluorescence images of spontaneous MMTV-PyMT tumour development. Staining for; cytokeratin-5 (K5; red), cytokeratin-8 (K8; green) and DAPI (blue). Representative images are shown for: (**a**) Ductal structures with K8 positive luminal cells surrounded by K5 positive basal cell lining, (**b**) hyperplasia, (**c**) adenoma-like growth and (**d**) carcinoma-like growth. Scale bar: 50 µm. (**e**–**f**) Immunoflourescence images of organoids from primary PyMT tumour cells (**e**) or primary normal mammary epithelial cells (**f**) cultured in matrigel/collagen gels for 2 weeks. Cultures are stained for cytokeratin-5 (K5; cyan), cytokeratin-14 (K14; red), cytokeratin-8 (K8; green) and DAPI (blue). Scale bar: 200 µm. (**g**–**i**) Haemotoxylin and Eosin (H&E) staining of different stages of tumourigenesis from 10-week old MMTV-PyMT Mammary gland (FVB). Representative images of (**g**) hyperplasia, (**h**) adenoma transition and (**i**) carcinoma. Scale bar: 50 µm. (**l**–**n**) H&E staining of primary PyMT tumour 2 weeks post-transplantation in growth factor reduced matrigel. Representative images show ductal-lobular structures within the centre of the plug (**l**); transition from a ductal-lobular structure to more tumour-like growth towards the edge of the plug (**m**); carcinoma-like growth at plug’s edge (**n**) Scale bar: 50 µm. (**o**–**p**) Ki67 DAB staining (left panel) and immunofluorescence staining for cytokeratin-5 (K5; red) and DAPI (right panel) of ductal-lobular structures (**o**) and tumour-like growth (**p**). Scale bar: 50 µm.

The mouse tumour model expressing Polyomavirus middle T oncogene (PyMT) under the control of the tissue specific mouse mammary tumour virus (MMTV) promoter (MMTV-PyMT), develops multifocal metastatic tumour in the mammary gland^22^. The expression of the viral oncogene in epithelial cells of the mammary gland leads to the multistage development of tumour, mimicking human tumour development and the global expression profile of tumours correlates with human disease^23^. The early stage starts with hyperplasia and adenomas that progress to carcinomas. Late carcinoma stage gives rise to spontaneous metastases to the lung^24^. PyMT tumours at the transition from the adenoma to the carcinoma stage break the basement membrane, the stroma surrounding epithelial cells increases and the presence of K5 myoepithelial cells starts to decrease to give rise to luminal K8 tumours (Fig. 1a–d and g–i). At the carcinoma stage cancer cells have undergone full malignant modifications with the development of a ER-negative phenotype and the over-expression of ErbB2^24^. Histologically, cells at the carcinoma stage display a highly unorganized growth with a dense stromal compartment (Fig. 1i). In line with early *in vitro* studies form Bissel’s group^5, 9, 10^, when cancer cells are isolated from late PyMT carcinomas and grown *ex vivo* in an ECM rich in collagen and basal lamina (matrigel/collagen), they adopt a “normalized” type of growth. Cells organize in mammary-like ducts and alveolar structures showing both K5 and K14 expression (Fig. 1e). Those structures resemble the one generated by normal primary mammary cells *ex vivo* grown in the same conditions (Fig. 1f). This type of growth is likely triggered by ECM-integrin signalling within this 3D *in vitro* environment^5, 10^. Interestingly, matrigel was shown to improve the efficiency of tumour transplantation^14^ and indeed when PyMT cancer cells from late carcinoma are transplanted into the fat pad of recipient mice, metastatic tumours are well recapitulated^25^. This tumour reconstitution can be very efficient and low number of cancer cells transplanted in matrigel onto recipient mice is the gold standard test to compare tumour initiation potential *in vivo* of different cancer cell sub-pools^2, 15, 16^. In order to investigate how the *in vitro* “normalizing” environment of matrigel impacts on early tumour growth *in vivo*, we analysed low number of transplanted cells in this ECM environment over time. One week after transplantation, similarly to the *in vitro* observations, primary PyMT cells adopt a similar ductal-lobular type of structures generated by primary normal mammary cells grafted in the same condition (Supp Fig. 1a and d). Clearly, cancer cells show more and bigger structures compared to normal cells, which are reminiscent of early stage of tumour hyperplasia (Fig. 1g,l). Over time the growth develops in two distinct and polarised types of growth: in the more central area of the plug cells in these hollow ductal-lobular structures are less proliferative and, similarly to the one formed by normal mammary cells, show K5 positive cells (Fig. 1l,o and Supp Fig. 1b and e). At the edges of the plug cells start adopting a highly proliferative tumour-like growth where K5 positive cells disappear (Fig. 1m,n and p and Supp Fig. 1b). Notably, alike the intermediate adenoma stage during spontaneous PyMT tumour formation, the reacquisition of tumour-like growth in transplanted tumours is concomitant of the host-derived stroma reconstitution (Fig. 1h,m). Indeed, the surrounding of the ductal-lobular structures in the more central area of the plug is dominated by ECM with few host infiltrating cells (Fig. 1l), suggesting that the tumour microenvironment support is required for tumour growth. After 3 weeks, PyMT cells and host derived tissue cells have completely filled the matrigel plug and fully reacquired the malignant and undifferentiated behaviour of the original cancer (Supp Fig. 1c). Collectively, these data show that cancer cells from a late stage metastatic carcinoma when transplanted in matrigel *in vivo*, are initially “rewired” to grow as differentiated ductal-lobular structures, but rapidly reacquire their aggressive behaviour as they gain access to host derived cells.

### Macrophages in the tumour microenvironment correlate with the reacquisition of cancer cell aggressive behaviour

**Figure 2:**
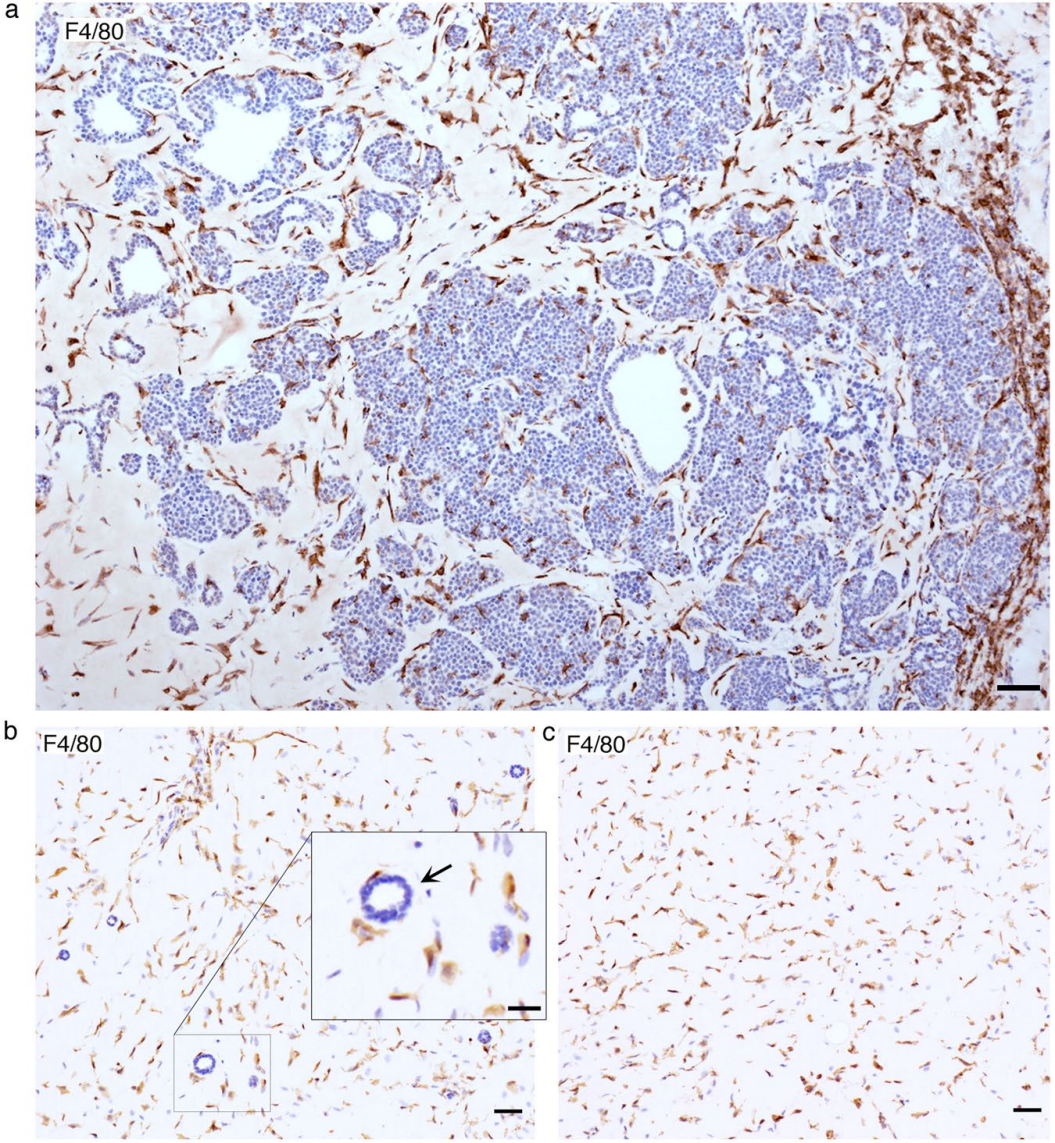
Analysis of stromal infiltrate in transplanted PyMT plugs. (**a**) Representative image macrophage staining (F4/80) at 2-week showing a transition from ductal- to a tumour-like growth with an increasing concentration of macrophages. Scale bar: 50 µm. (**b**) Representative image macrophage staining (F4/80) of a primary normal mammary cell transplant at 1-week. Scale bar: 50 µm. Insert close up of a cross-section of a normal duct and surrounding F4/80 positive macrophages, Scale bar: 25 µm. (**c**) Representative image macrophage staining (F4/80) of an empty Matrigel plug 1-week post-transplantation. Scale bar: 50 µm.

Given the clear correlation between the reacquisition of the cancer cell malignant phenotype and the reconstitution of the tumour associated host microenvironment, we next investigated the different host cells infiltrating the plug over time. As summarized in Supplementary Table 1, adaptive immune cells (T and B lymphocytes) are sporadically present. Also, endothelial cells are not present within the plug at early stages, but significantly increase at later stages of tumour growth (Supp Table 1, Supp Fig. 2a). Contractile activated fibroblasts, labelled by alpha smooth muscle actin (αSMA), start infiltrating the plug early and increase their presence over time to become one of the dominant cellular components of the tumour microenvironment at the late stage of tumour growth (Supp Table 1 and Supp Fig. 2b). Strikingly, the host component showing the stronger presence early on within the matrigel plug are F4/80 positive macrophages (Supp Table 1 and Supp Fig. 2c). During the intermediate stage, where the two types of growth are observed in the centre and at the edges of the matrigel plug, macrophages also show a higher concentration surrounding the tumour-like type of growth (Fig. 2a). Interestingly the early macrophage infiltration seems to be triggered by the presence of the matrigel plug and not specifically by cancer cells, as macrophages are also the main host cell infiltrate observed when normal primary mammary epithelial cells are transplanted or even in empty matrigel plugs (Fig. 2b,c). However, a similar high macrophage presence is observed during the early phases of spontaneous PyMT tumour development (Supp Fig. 3), suggesting that they are also part of the endogenous response of the tissue to tumour development.

### Macrophages infiltrating matrigel plugs are crucial for the reacquisition of carcinoma growth *in vivo*

**Figure 3:**
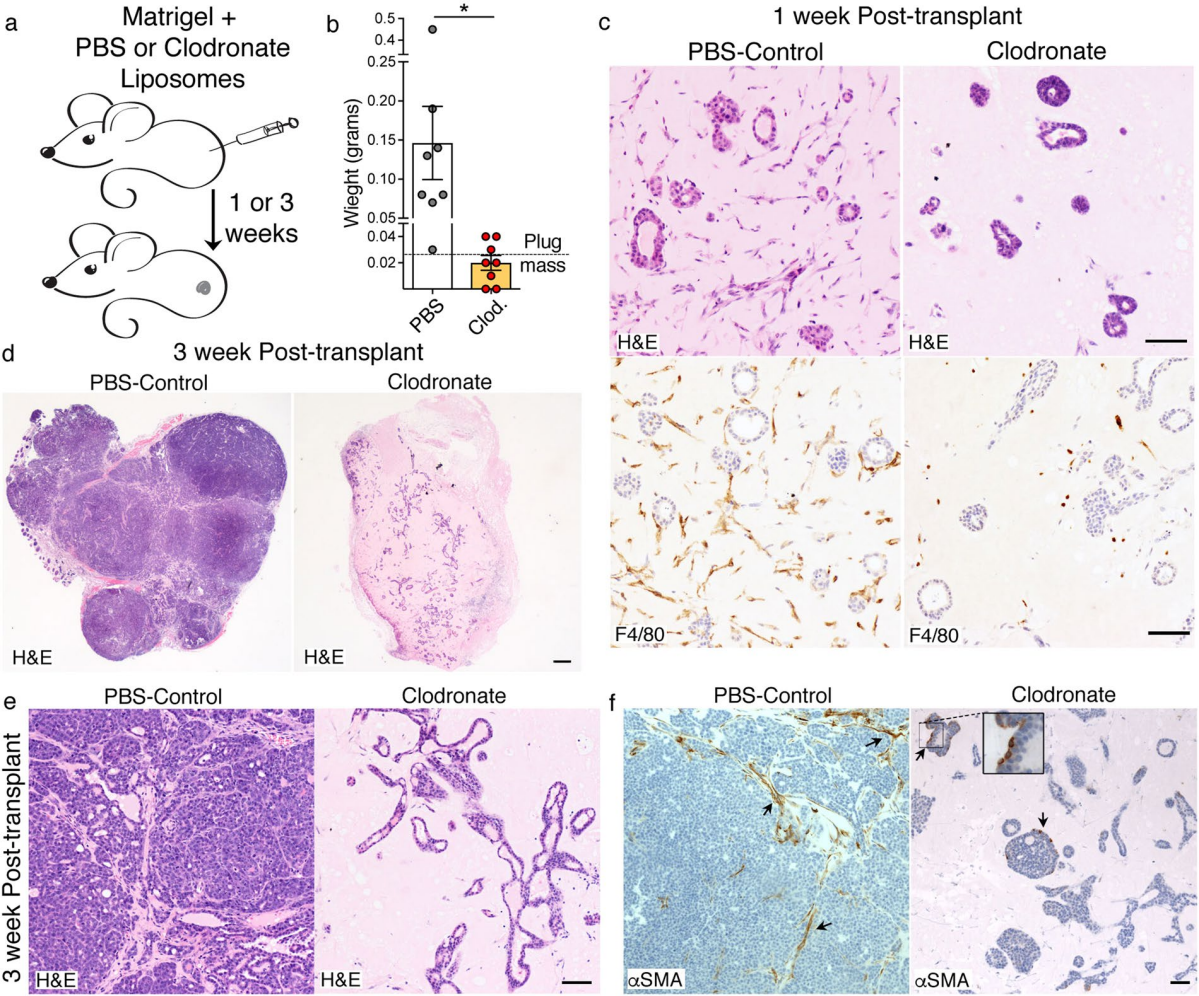
Blocking macrophages prevents outgrowth of PyMT tumour cells. (**a**) Experimental design used to block macrophages during transplantation. (**b**) Plug mass 3 weeks after PyMT transplantation in presence in either PBS or Clodronate (Clod) liposomes. *P value < 0.05 (n = 7, two tail T-test). (**c**) H&E (Upper panels) and immunohistochemical staining for F4/80 (Lower panels) 1 week post-transplantation in growth factor reduced matrigel with either PBS or Clodronate. Scale bar: 50 µm. (**d**,**e**) H&E of staining of PBS or Clodronate treated plugs at 3-week post-transplantation. Scale bar: 200 µm (**d**); Scale bar: 50 µm (**e**). (**f**) Immunohistochemical staining for αSMA of PBS or Clodronate treated plugs 1 week post-transplantation. Scale bar: 50 µm.

Considering the strong correlation between the reacquisition of the advanced cancer-type of growth together with their well accepted crucial role as driver of tumour promoting inflammation^17^, we tested if the macrophage presence in the matrigel environment is important for the cancer cell growth behaviour. We used Clodronate liposomes, which selectively kill phagocytic cells and are extensively used to deplete macrophages^26^. PyMT cells in matrigel plugs were injected in recipient mice that were systemically and locally treated with Clodronate or PBS control liposomes (Fig. 3a). Plugs harvested after 1 week confirmed macrophage depletion (Fig. 3c). At this stage, the ductal-lobular cancer cell growth is not influenced by macrophage depletion. Strikingly, at the 3-week time point when tumour growth is reconstituted in the control plugs, tumours fail to develop in absence of macrophages (Fig. 3b and d). Histology of Clodronate treated plugs shows cancer cells growing in a *matrigel-segregated* environment and forming widespread ductal-lobular type of growth (Fig. 3e right panel). Conversely, cancer cells in control plugs recapitulate their aggressive phenotype and reconstitute their host associated stroma environment (Fig. 3e, left panel). Notably, depletion of macrophage from the plug environment also has the effect of preventing other host cells infiltration. While control transplants grow tumours rich in contractile αSMA-positive fibroblasts, in macrophage-depleted plugs, the only cells positive for αSMA are some myoepithelial cells of the ductal-lobular structures, which share the expression of this marker with activated fibroblast (Fig. 3f).

### Macrophages infiltrating the plug adopt a mesenchymal phenotype promoting ECM degradation

**Figure 4:**
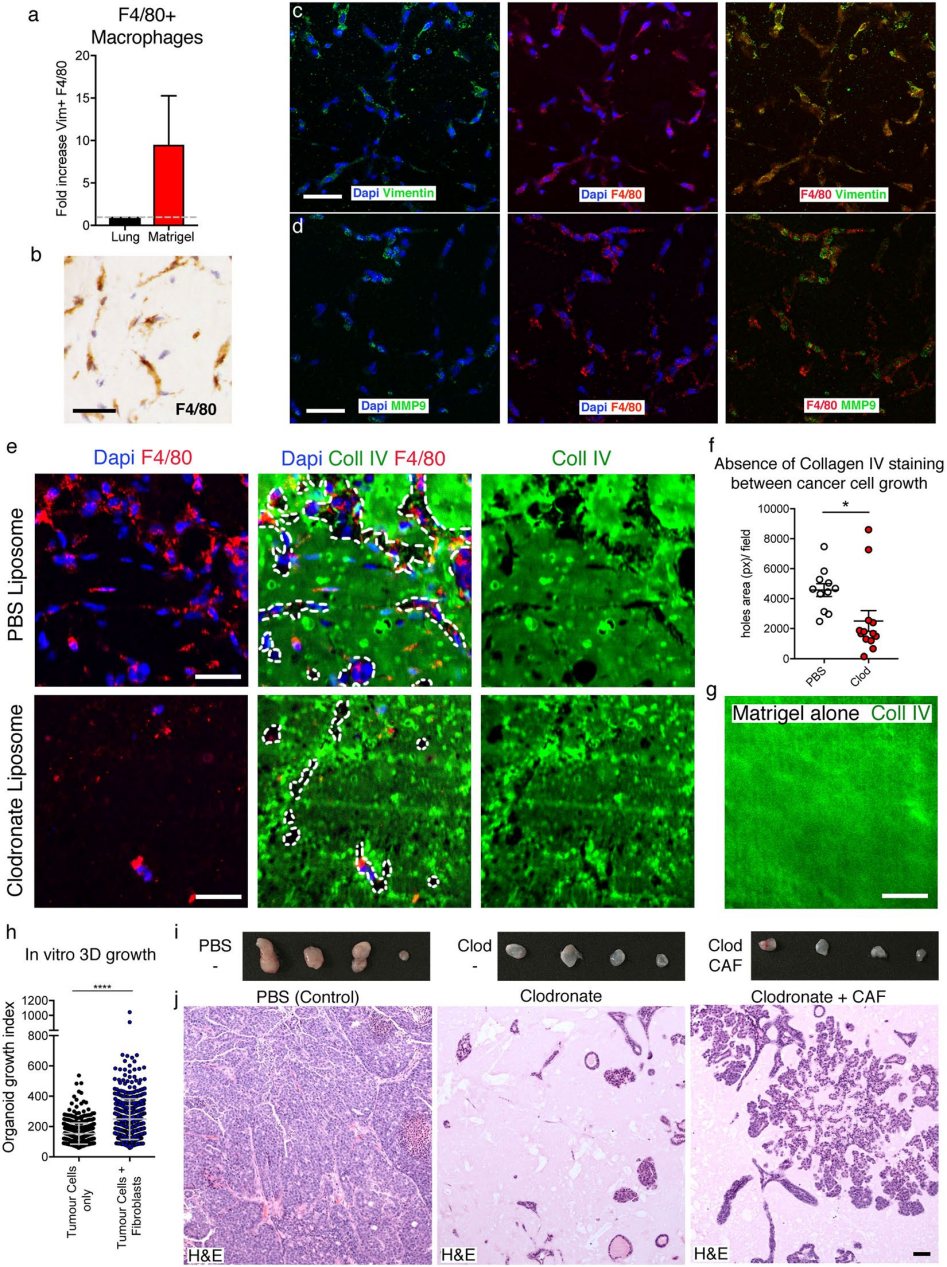
Plug macrophages show mesenchymal feature and remodel the matrigel. (**a**) Intracellular FACS analysis for vimentin in F4/80+ macrophages isolated from growth factor reduced matrigel plug 1-week post-transplantation or lungs. Data from three independent experiments. (**b**–**d**) Immunofluorescence or Immunohistochemical staining of PyMT plugs harvested 1 week post-transplantation in growth factor reduced matrigel. Representative immunohistochemical staining for F4/80 (**b**). Immunofluorescence staining for vimentin (**c**) or MMP9 (**d**) (green), F4/80 (red) and DAPI (blue). Scale bar: 50 µm. (**e**) Representative immunofluorescence staining for collagen IV (green), F4/80 (red) and DAPI (blue) of a representative field of matrix either in PBS-control plug (upper panels) or Clodronate Plug (lower panels). White lines show absence of Collagen IV. Scale bar: 50 µm. (**f**) Quantification of total lack of collagen IV staining. *P value < 0.05 (n = 4, two tail T-test). (**g**) Representative collagen IV staining in an intact cell-free growth factor reduced matrigel plug generated *in vitro*. (**h**) Quantification of organoids from PyMT tumour cells (TC) grown with or without addition of a fibroblast cell-line (T50). (**i**) Photos of explanted PyMT transplanted either in presence of PBS, Clodronate or Clodronate and a cancer-associated fibroblasts (CAF) cell line 3-week post-transplantation. (n = 4) (**j**) H&E PyMT transplanted either in presence of PBS, Clodronate or Clodronate and a cancer-associated fibroblasts (CAF) cell line in a 1:1 ratio at 3 weeks. Scale bar: 50 µm.

Considering the persistence of the *matrigel-segregation* effect observed in transplants where macrophages are depleted, we analysed how macrophages infiltrate the three-dimensional (3D) environment of matrigel *in vivo*. Macrophages are known to adopt a different type of migration accordingly to the ECM composition, an ameboid migration in presence of fibrillary collagen or mesenchymal migration in stiffer matrixes such as matrigel^27^. Mesenchymal migration strongly depends on the secretion of proteases leading to ECM degradation. Indeed, macrophages infiltrating matrigel plugs display elongated cell shape with protrusions and ﻿the﻿ expression of the mesenchymal markers Vimentin and metalloprotein MMP9 (Fig. 4a–d). Notably, large areas of the ECM are missing surrounding macrophage clusters as shown by the lack of staining for Laminin and Collagen IV, the main matrigel components (Fig. 4e–g and Supp Fig. 4a). When staining for Collagen IV within control plugs, where macrophages can infiltrate, larger areas lacking staining are present in the matrix between the cancer cell growth in comparison to Clodronate plugs (Fig. 4e, f). Moreover, a direct correlation between those Collagen IV or laminin dark areas and the presence of macrophages can be observed (Fig. 4e and Supp Fig. 4a). Importantly, those basal lamina ECMs seem to be the main driver of macrophage recruitment as an increase in growth factor concentration in matrigel does not boost early macrophage infiltration (Supp Fig. 4b, c). Additionally, Collagen I plugs elicit macrophage recruitment, but display less infiltration within the matrix core (Supp Fig. 4d). This evidence suggests that macrophages, at this early time point of transplantation are attracted mainly by the ECM components of the matrigel plugs, and induce their degradation. Therefore, in addition to other described activities of macrophages to promote tumour growth and progression when interacting with cancer cells, in this experimental setting, one important function of macrophages is to disrupt matrigel surrounding cancer cells suffering *matrigel-segregation* to allow other host cells to interact with cancer cells. Indeed, Clodronate treated plugs lack any other host cells (Fig. 3c–e) including αSMA positive fibroblasts, the main component of tumour associated stroma and normally infiltrating matrigel since an early time point (Supp Table 1, Supp Fig. 2b and Fig. 3f, left panel).

Fibroblasts boost growth of PyMT cells in matrigel culture *in vitro* (Fig. 4h), yet, adding CAFs to Clodronate-treated plugs in a similar amount as observed in early stages of transplantation (Supp Fig. 2b), did not rescue cancer growth after 3 weeks (Fig. 4i). It has been demonstrated that an evolution of the CAFs characteristics within the tumour microenvironment is required during tumour progression^28^. Indeed, histologic analysis shows that the level of cancer cell growth within the plugs *in vivo* is enhanced by the presence of CAFs, but the further infiltration of other cells including host derived fibroblasts does not occurs preventing the required complex tumour microenvironment reconstitution (Fig. 4j).

These data support the idea that one of the crucial roles of macrophages in the PyMT cancer cell transplantation assay is to act as gatekeepers of the tumour microenvironment, the required structure allowing tumour growth. Notably, by preventing macrophage infiltration, cancer cells are maintained within the matrigel plug isolated from the rest of the host tissue, where this ECM environment forces them to maintain a low proliferating and differentiated type of growth, which is likely less capable of attracting host derive cells such as fibroblasts.

### Cancer cells in macrophage-deprived plugs maintain their full tumour initiating potential

**Figure 5:**
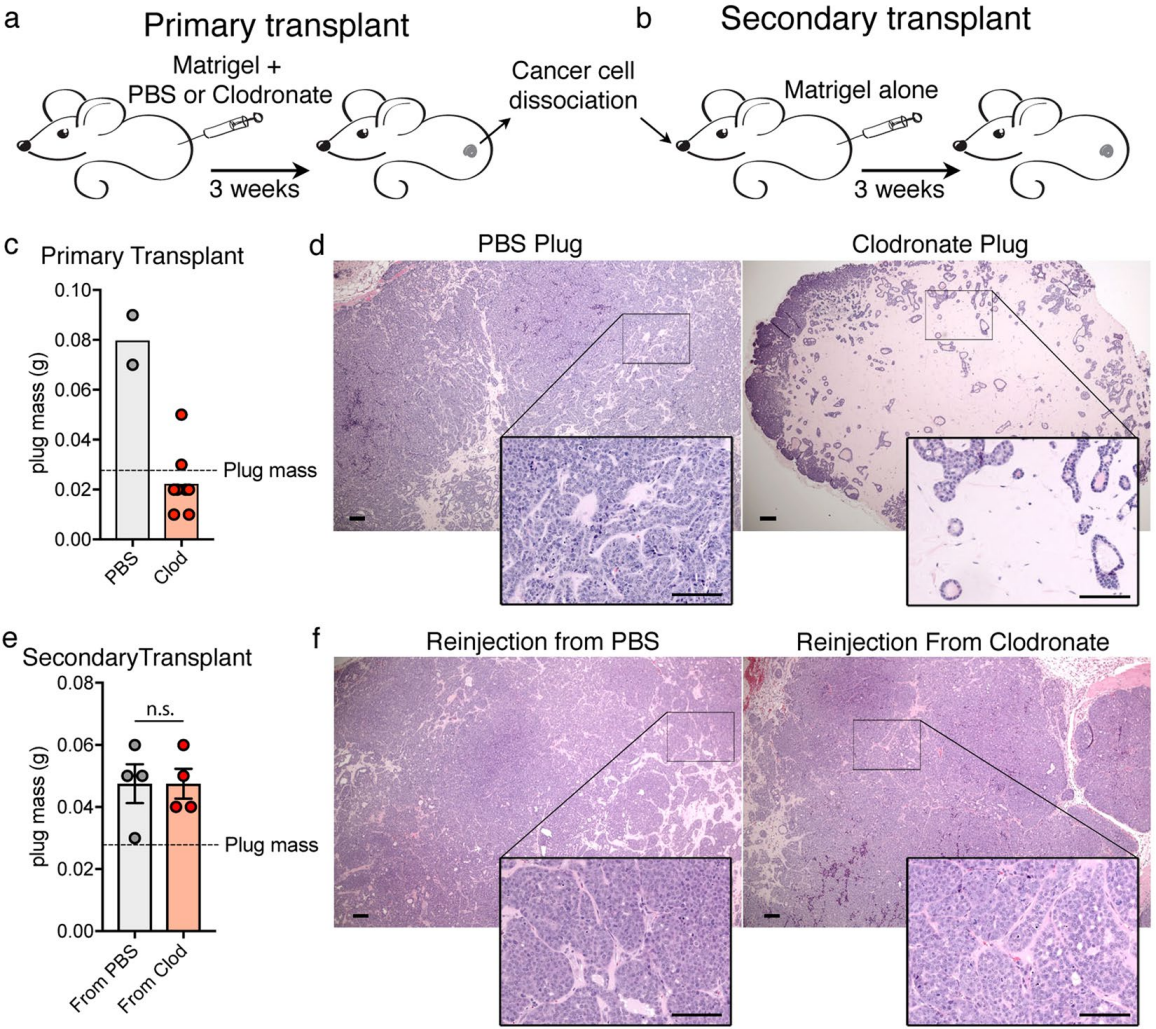
PyMT cells in clodronate treated Matrigel retain intrinsic ability to form tumour. (**a**) Experimental design for primary transplant, in presence of either PBS or Clodronate liposomes. (**b**) After 3 weeks secondary transplants in naïve mice. (**c**) Plug mass of primary transplant 3 weeks post-transplantation in growth factor reduced matrigel. (PBS n = 2, Clodronate n = 6). (**d**) H&E of PBS or Clodronate treated plugs at 3 weeks post-transplantation. Scale bar: 100 µm. Inset: higher magnification. Scale bar: 100 µm. (**e**) Plug mass of secondary transplants 2.5 weeks post-transplantation in Matrigel only. (n = 4, n.s. two tail T-test non-significant). (**f**) H&E of PBS or Clodronate treated plugs at 2.5 weeks post-transplantation. Scale bar: 100 µm. Inset: higher magnification. Scale bar: 100 µm.

We next tested whether cancer cells lose their tumorigenic potential upon prolonged growth *in vivo* in presence of Clodronate. Cancer cells were harvested either from tumours outgrown in PBS control plugs or from normalized lobular structures grown in Clodronate plugs (Fig. 5a–d). Low number of cancer cells from each preparation were tested in secondary transplants. Equal number of cancer cells were reinjected in new recipient mice in absence of Clodronate (Fig. 5b). Remarkably, similar tumour reconstitution was observed in both secondary transplant groups (Fig. 5e,f) indicating that cancer cells maintain their tumour initiating potential intact, which was conditionally repressed *in vivo* by the matrigel context and the absence of tumour host interactions.

### Diverse breast cancer cells differently depend on macrophages in matrigel transplantation assays

**Figure 6:**
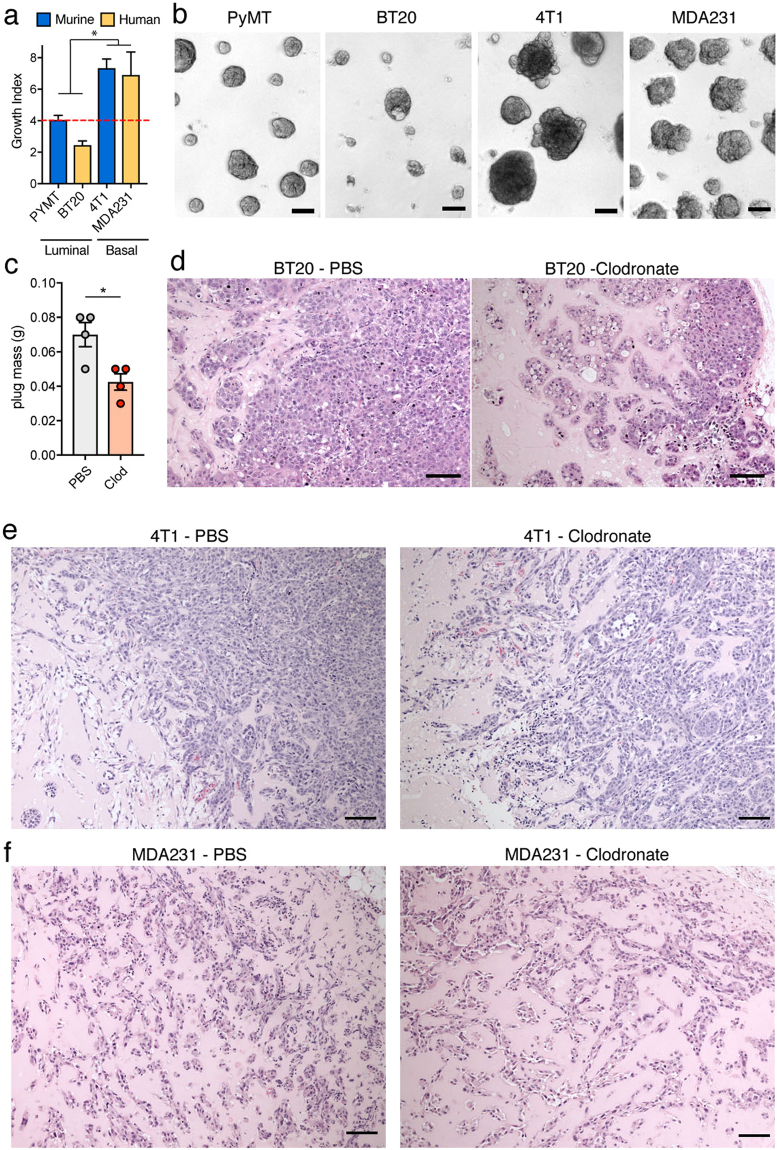
Different lines grow differently in Matrigel *in vitro* and differently require macrophages *in vivo*. (**a**) Quantification of the indicated cancer cell lines growth *in vitro* on growth factor reduced matrigel. *P value < 0.05 (two-way ANOVA between the growth of luminal vs basal cells) (**b**) Bright field images of the indicated cancer cell lines on matrigel. Scale bar: 100 µm. (**c**) Mass of BT20 transplanted and growth for 7 weeks either with PBS or Clodronate liposomes in growth factor reduced matrigel. *P value < 0.05 (n = 4, two tail T-test) (**d**) H&E of BT20 tumours in c. Scale bar: 100 µm. (**e**) H&E of 4T1 transplanted grown for 1 week either with PBS or Clodronate liposomes in growth factor reduced matrigel. Scale bar: 100 µm. (**F)** H&E of MDAMB231 transplanted grown for 1 week either with PBS or Clodronate liposomes in growth factor reduced matrigel. Scale bar: 100 µm.

Breast cancer can resemble either the luminal or the basal layer of mammary duct. Cancer cells from these different types have distinctive characteristics: basal types of breast cancer have a mesenchymal phenotype and are oestrogen receptor negative. When tested for their *in vitro* ability to colonize matrigel, the basal murine cell lines 4T1 and the human MDA-MB-231 showed an increased growth compared to luminal cancer cell types, the murine PyMT and human BT-20 cells (Fig. 6a,b). This suggests that their diverse characteristics result in a different susceptibility to the ECM environment of matrigel. Interestingly, in correlation with their reduced ability to grow *in vitro* on matrigel compare to the 4T1 and MDA-MB-231 cells, BT-20 cells show a decreased growth when transplanted *in vivo* in presence of Clodronate (Fig. 6c). Histology of Clodronate-treated plugs shows that BT-20 growing in matrigel, similarly to PyMT cells, suffer of *matrigel-segregation* and adopt a more polarized phenotype compare to controls cells, which were able to form tumours more efficiently (Fig. 6d). This suggests that, BT-20 cells also rely on macrophage infiltration to efficiently gain access to the host cells and to interfere with the inhibition imposed by the matrigel ECM. In contrast, the tested basal cancer cell lines, are not affected by the absence of macrophages *in vivo* and their mesenchymal phenotype and invasive modality of growth is maintained within Clodronate-treated matrigel plugs, suggesting a reduced impact of this ECM context on their growth in this transplantation setting (Fig. 6e,f). Interestingly, the tested basal cancer cell lines also seem to trigger a macrophage reaction only at the edge of the matrigel plug and an absence of overall macrophage infiltration, suggesting that their different growth behaviour also has consequences for cancer cells-macrophage interactions (Supp Fig. 5).

## Discussion

Tumour transplantation methods are a very common strategy to propagate primary tumours from animal to animal or to perform various experimental settings either in syngeneic models or in immune-deficient recipients. The use of basement membrane matrix proteins, such as matrigel, was proven to increase initiation and growth of tumour cell lines, cancer stem cells and primary cancer cells in recipient mice^14^. This is now a commonly used protocol *in vivo* tumour biology studies, including testing tumour initiation potential in limiting dilution assays^2, 15, 16^. Notably, it is long known that ECM components can lead to changes in the gene expression influencing cells’ phenotype^11^. Indeed, important factors in the tumour microenvironment allowing abnormal cell behaviour, are changes in the ECM composition and stiffness with altered basement membranes, which is normally separating the epithelial from the connective tissue^13^. The impact of the ECM and its receptors dictates the phenotype of breast cancer cells, and *in vitro* ECM system has demonstrated that the phenotype of malignant cancer cells can be modified by extracellular signals^10^. However, the impact of transplanting breast cancer cells embedded in basement membrane matrix proteins such as matrigel on their initial phenotype and growth was never investigated as well as the important events required for tumour reconstitution.

In this study, we followed the growth over time of low number of cancer cells isolated from advance MMTV-Polyoma Middle T antigen (PyMT) mouse breast cancers to assess how early growth is influenced by the presence of basement membrane matrix proteins and the events influencing the reacquisition of their aggressive phenotype. We found that, similarly to the normalized growth adopted in a three-dimensional (3D) *ex vivo* culture, also their early *in vivo* growth within matrigel plugs resembles a differentiated ductal-lobular structure organized in luminal epithelial cells surrounded by basal-like cells. This emphasizes the potential of the ECM to re-program the aggressive phenotype of breast cancer cells *in vivo*. This inhibitory environment initially generates cancer cell *matrigel-segregation*. This effect is likely caused by a vicious cycle triggered by the matrigel “normalizing” effect on the transplanted cancer cells, that, alongside the low aggressive behaviour, is likely causing a decrease of the tumour-derived chemokines released that would normally trigger attraction of host cells. However, *in vivo* despite this initial isolated environment, the interaction with host cells still has the potential to modify the surrounding of cancer cells. Particularly we found that early macrophage infiltration, mainly elicited by the same matrigel components responsible for the *matrigel-segregation* effect, produces its degradation. Indeed, by using Clodronate liposome treatment to prevent macrophage infiltration, we achieved long term *matrigel-segregation* of metastatic PyMT cells within this normalizing environment and failure of tumour reconstitution. This prolonged normalized phenotype of the transplanted cancer cells, likely impairs their ability to attract of host cells, such as of αSMA positive fibroblast. This could explain the absence of any host cells observed in macrophage deprived plugs. Certainly, many pro-tumorigenic activities of tumour associated macrophages have been characterized^18^, and their absence within the tumour microenvironment will likely have a negative impact on other aspects of the tumorigenic behaviour of cancer cells. Particularly the direct interaction between macrophages and cancer cells reacquiring an aggressive behaviour at the edges of the matrigel plug, will likely directly boosts tumour growth. However, this study points at an additional important function of macrophages as gatekeepers of tumour host interactions in this matrigel transplantation experimental setting.

Importantly, when cancer cells, such as basal-like cells, are able to maintain an invasive behaviour within the matrigel environment, they are likely more self-sufficient in gaining access to host-derived cells and consequently do not rely on macrophage presence. Interestingly, this different cancer cell growth behaviour is also reflected in a reduction of macrophage presence, suggesting a differential cancer cells-macrophage crosstalk at this early stage of tumour growth within the matrigel plug.

Remarkably, the intrinsic tumorigenic potential of PyMT cells forming ductal-lobular structures *in vivo* remains unaltered and they can give rise to tumours in secondary transplants. These data show that a big part of the tumorigenic potential of PyMT cells is to control the surrounding tissue environment and the ability to reconstitute their host-derived supportive structure. Crucially, this also represents a decisive proof of the tumour microenvironment’s requirement for the maintenance of cancer cells malignant behaviour and its possible overruling power over the intrinsic tumorigenicity provided by oncogenic pathways.

Notably, as different breast cancer cell types are differently affected by matrigel in their growth, also different sub-pools of cancer cells within the same tumour displaying different phenotypic characteristics, might be differently influenced by matrigel. In conclusion, both extrinsic factors and the interaction with the ECM components of matrigel need to be taken into consideration when testing breast cancer cells’ tumour initiation ability in transplantation assay before drawing conclusions on their intrinsic tumorigenicity.

## Materials and Methods

### Animal strains

Transgenic FVB/n mice expressing the Polyoma Middle T-antigen (PyMT) oncogene under the Mouse Mammary Tumor Virus promoter (MMTV-PyMT), used as sources of primary cancer cells, were originally obtained from Jax Laboratory and breed within our establishment for many years. Cells were isolated from late stage carcinomas.

RAG1 KO FVB/n mice were kind gifts from Dr Joerg Huelsken (EPFL, Lausanne). Age matched 6 to 10 week old females were used as recipients for all tumour transplantations. Breeding and all animal procedures were performed at our establishment in accordance with UK Home Office regulations under project license PPL number P83B37B3C. The NCRI Guidelines for the Welfare and Use of Animals in Cancer Research were strictly followed.

### Animal Experiments

In tumour transplantation assays, either 1 × 10^4^ PyMT primary cells, 1 × 10^4^ BT20, 1 × 10^4^ 4T1 or 1 × 10^6^ MDAMB231 cells were mixed in 50 μl of Growth Factor Reduced Matrigel (Corning), and injected subcutaneously into the flanks of anesthetized mice. Matrigel injection was performed either orthotopically in the mammary fad pad or sub-cutaneous. As the results on host infiltration in the matrigel plugs were the same in the two settings the majority of the experiments were performed sub-cutaneous to allow better recovery of the matrigel plugs. The same type of cell infiltrates were also observed when using WT or RAG1 ko animals at the early stage of this experimental setting. For macrophage blocking experiments 20 μl of PBS or Clodronate liposomes (clodronateliposomes.com) were subcutaneously injected on the flank immediately prior to injection of cells. The mice were also given an intraperitoneal injection of 150 μl PBS or Clodronate liposomes twice weekly. For the co-injection of PyMT cells and CAFs, a 1:1 ratio was used: 1 × 10^4^ PyMT primary cells and 1 × 10^4^ CAFs.

### Histology

Tumour plugs were fixed in 10% NBF for 24 h and embedded in paraffin blocks. Four-micron sections were stained. For immunohistochemistry, secondary biotin-conjugated antibodies were used in combination the VECTASTAIN ABC kit (all Vector Laboratories) according to the manufacturer’s instructions. When immunofluorescence staining was used tissue was treated the same except secondary alexafluor-conjugated antibodies were used. Specific primary antibodies were used (table) visualization of cell nuclei was performed with haematoxylin or DAPI. Antibodies: F4/80 (14-4801, eBioscience); Cytokeratin-5 (K5) (ab24647, Abcam); Cytokertain-8 (K8) (ab_531826, DSHB); Cytokeratin-14 (ab7800, Abcam); Endomucin (sc65495, Santa Cruz); LYVE-1 (ab33682, Abcam); Ki67 (ab16667, Abcam); αSMA (A2547, Sigma); Vimentin (V2258, Sigma); Laminin (ab11575, Abcam); Collagen IV (ab6586, Abcam); MMP9 (ab38898, Abcam); S100A9 (Crick in House produced); CD3 (ab134096, Abcam); B220 (553086, BD Pharmingen).

Images were quantified using ImageJ. For the lack of Collagen IV quantification, smaller fields in four 10x images were selected in order to avoid areas of direct cancer cell growth. The wand tool was used to select all the black areas in each field in the Collagen IV channel of the immunofluorescence staining. The total Pixels area was quantified for each field. For the quantification of the F4/80 staining within the matrigel plugs, the “Analyse Particles” function was used on the selected plug area of 4x immunohistochemistry images sections.

### Cell Culture

Mouse mammary carcinoma PyMT cells were seeded 2D in plates coated with collagen-solution (100 mg/ml BSA, 1 M HEPES, 1:100 PureCol (Advanced BioMatrix) in HBSS), and maintained in MEM media (DMEM/F12 supplemented with 2% FCS, 10 μg/ml Insulin (Sigma), 20 ng/ml EGF (Invitrogen) and 1:50 L-Glutamax (Life Technologies)), unless otherwise specified, and grown at 37 C and 5% CO_2_. The fibroblast cell-line T-50 was generated by immortalising normal mammary gland fibroblast from a FVB/n MMTV-PyMT negative female and CAFs by immortalising fibroblasts from carcinoma from a FVB/n MMTV-PyMT positive female both according to the published bio-protocol e1097^29^ (http://www.bio-protocol.org/e1097). BT20 was maintained in EMEM + 10% FCS whilst 4T1 and MDAMB231 were maintained in DMEM (Gibco) + 10% FCS and grown at 37 C and 5% CO_2_. Cell lines were obtained from Crick Cell Services STP and undergo STR typing and mycoplasma testing.

### Tissue Digestion

MMTV-PyMT cell isolation was described in detail previously. In brief, primary MMTV-PyMT tumours were dissected, minced, and digested with Liberase (Roche) and DNaseI (Sigma) in HBSS and passed through a 100 μm cell strainer. For reinjection experiments and FACs analysis of macrophages, primary transplant matrigel plugs were digested with dispase at 2 U/ml at 37 C for 1–2 hours and either stained immediately (FACS) or plated overnight on collagen-coated plates before the secondary transplant.

### 3D Assays

Tumour plugs were fixed in 10% NBF for 24 h and embedded in paraffin blocks. Four-A single cell suspension of GFP^+^ PyMT primary tumour cells were directly seeded on a glass-bottom 35 mm MatTek dish (MatTek) at 1 × 10^4^ cell/dish in a 2:1 mixture of collagen-I (BD Biosciences) and Matrigel (BD Biosciences), yielding a final collagen concentration of ~4.6 mg ml^−1^ and a final Matrigel concentration of ~2.2 mg ml^−1^, and maintained in MEM media at 37 C and 5% CO_2_ for 10 days. Normal fibroblast cell-line T50 were added to the gel at a ratio of 1:1 against tumour cells where specified. Organoids were imaged with an inverted Nikon ECLIPSE TE2000-E whilst image processing and analysis was performed with MetaMorph software (Molecular Devices Corp.). The organoid growth was quantified as the total GFP area per field of view. The degree of branching was manually quantified as percentages of spheres and branched growths.

Sphere formation on matrigel was performed by polymerizing a layer of growth factor reduced matrigel (Corning) into 24 well plates (Costar) at 37 C and seeding cells directly on top at density of 30,000 per well. Media was refreshed every 2–3 days. The wells were imaged after 6 days in culture using an EVOS microscope (Thermo Fisher). Analysis was performed using ImageJ, quantifying the total growth (index of both size of spheres and number) as a relation to the area of the field.

### Flow Cytometry and Sorting

For intracellular staining of macrophages for vimentin, tissue was digested as described to prepare a single-cell suspension. The preparation was incubated with LIVE/DEAD stain Zombie Violet before being incubation with FcR Blocking Reagent followed by extracellular staining with CD11b-FITC and Ly6G-PE. The preparations were then fixed and permeabised before staining with Vimentin-APC. The LSRFortessa cell analyser running FACSDiva software (BD Biosciences) and FlowJo software was used. Prepared single-cell suspensions of PyMT tumour preparations were incubated with mouse FcR Blocking Reagent (Miltenyi) followed by incubation with CD24-e450, CD45-PE, CD31-PE, Ter119-PE, AXL-biotin (in combination with Streptavidin-APC780). Dead cells were stained with or propidium iodide (PI; Sigma). Tumour cells were flow-sorted using the Influx cell sorter running FACS Sortware sorter software (BD Biosciences).

### Statistical Analysis

Data analyses used GraphPad Prism version 7. Two tail unpaired Student’s *t* test was used to determine statistical significance for all the panels displayed with the exception of data in Supplementary Figure 4 were Two-way ANOVA was used.

P value: *P < *0.05*, **P < 0.01, ***P < 0.001, ****P < *0.0001*, n.s. not significant.

## Electronic supplementary material

Supplementary Figures & legends
