# Supplementary material for: Macrophages unlock progression of breast cancer cells experiencing matrigel-segregation in transplantation models
Source: Sci Rep. 2017 Sep 8;7:11028. doi: 10.1038/s41598-017-11403-w (PMC5591247; doi:10.1038/s41598-017-11403-w)
Supplement: Supplementary file 1 — Supplementary Figures & legends [file 41598_2017_11403_MOESM1_ESM.pdf]

Title:

Macrophages unlock progression of breast cancer cells experiencing *matrigel-segregation* in transplantation models

Authors:

Misa Ogura[1]*, Victoria L. Bridgeman[1]*, Ilaria Malanchi[1#]

Tumour Host Interaction Lab, The Francis Crick Institute, 1 Midland Rd, NW1 1AT London, UK.

* These authors have contributed equally to this work.

# Corresponding author, Ilaria.Malanchi@crick.ac.uk.

Supplementary Figure and Legend

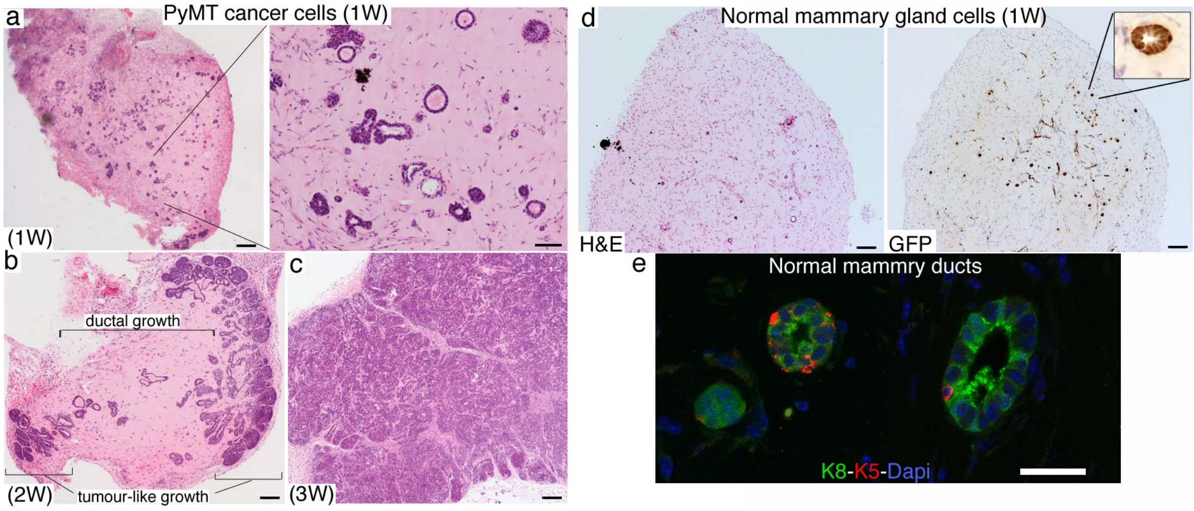

Supplementary Figure 1. Development of PyMT transplants over time
a-d H&E of PyMT transplants at 1 week (**a**), 2 weeks (**b**) or 3 weeks (**c**) post-transplantation in growth factor reduced matrigel. Scale bar: 200 μm. Inset: close up of 1 week PyMT transplant. Scale bar: 50 μm. **d** H&E (left panel) and immunohistochemistry for GFP staining (right panel) of primary normal mammary epithelial cells isolated from actin GFP+ transgenic mice at 1 week post-transplantation. **c** Immunofluorescence staining for cytokeratin-8 (K8; green), cytokeratin-5 (K5; red) and DAPI (blue) of normal ducts formed in primary normal mammary epithelial cells transplants after 1 week. Scale bar: 20 μm.

Table of host infiltrating components

Cell type	1W	2W	3W
αSMA Fibroblasts	+/-	+	+++
Endomucin Endothelial	-	+	++
Lyve-1 Lymphatic	ND	ND	ND
F4/80 Macrophages	+++	+++	++
S100A9 Neutrophils	ND	ND	ND
CD3 T Cells	+/-	+/-	ND
B220 B cells	ND	ND	ND

Supplementary Table 1. Analysis of stromal infiltrate in transplanted PyMT plugs

Summary of host-infiltrating cells in tumour transplants harvested at week 1, 2 or 3 post-transplantation in growth factor reduced matrigel analysed by the indicated immunohistochemical staining.

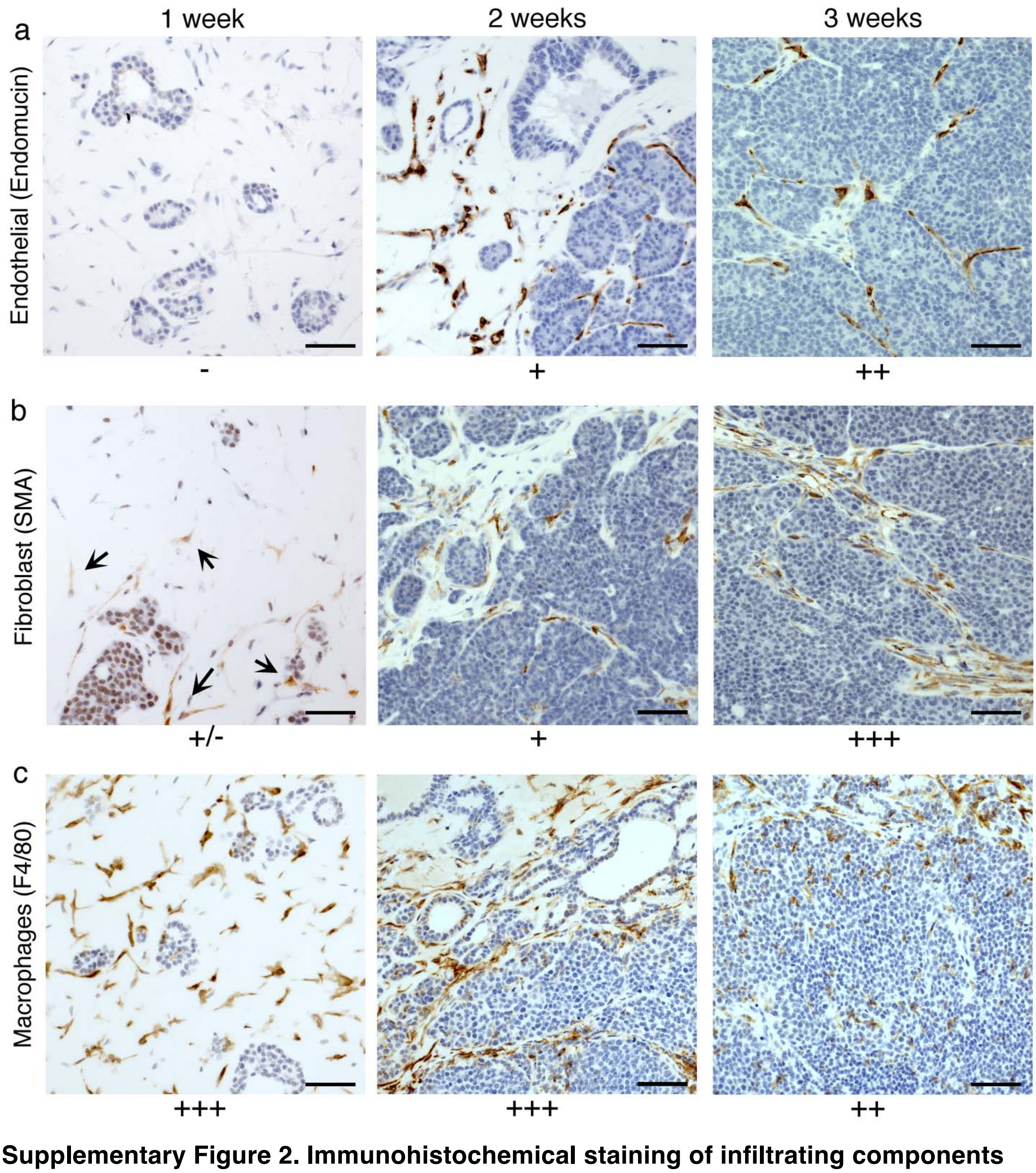

Supplementary Figure 2. Immunohistochemical staining of infiltrating components

Immunohistochemical staining of PyMT transplants harvested at 1, 2 or 3 weeks post-transplantation in growth factor reduced matrigel and stained for **a** endomucin (endothelial cells), **b** alpha Smooth Muscle Actin (αSMA) (arrow at 1 week time point indicate positive fibroblasts) and **c** F4/80 (macrophages). Scale bar: 50 μm

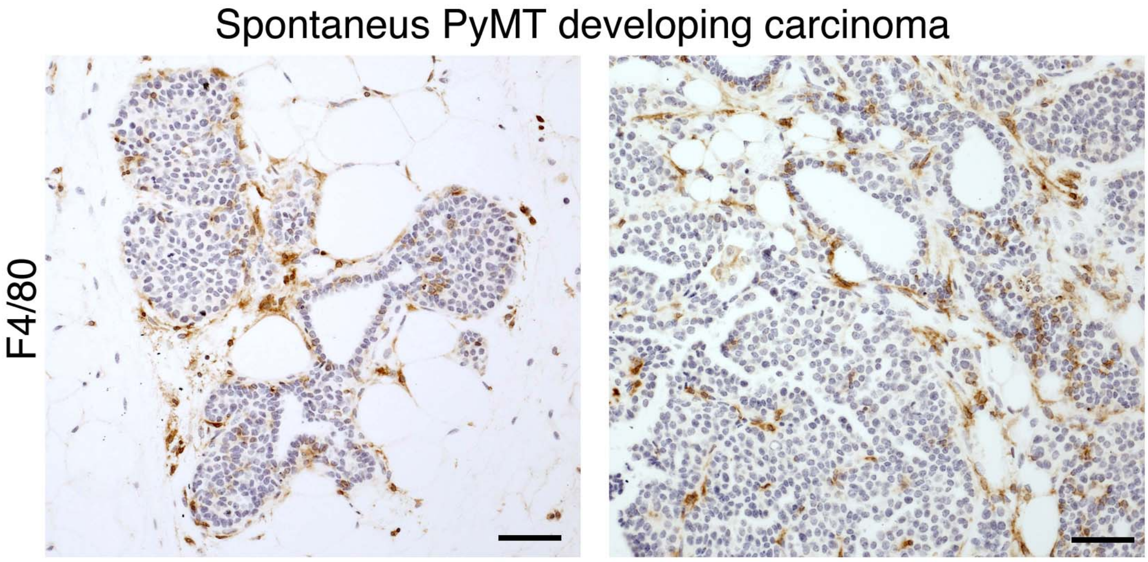

Supplementary Figure 3. Macrophage infiltration into spontaneous PyMT tumours

Immunohistochemical staining for F4/80 (macrophages) in spontaneous PyMT tumours formed in female MMTV-PyMT mice. Scale bar: 50 μm

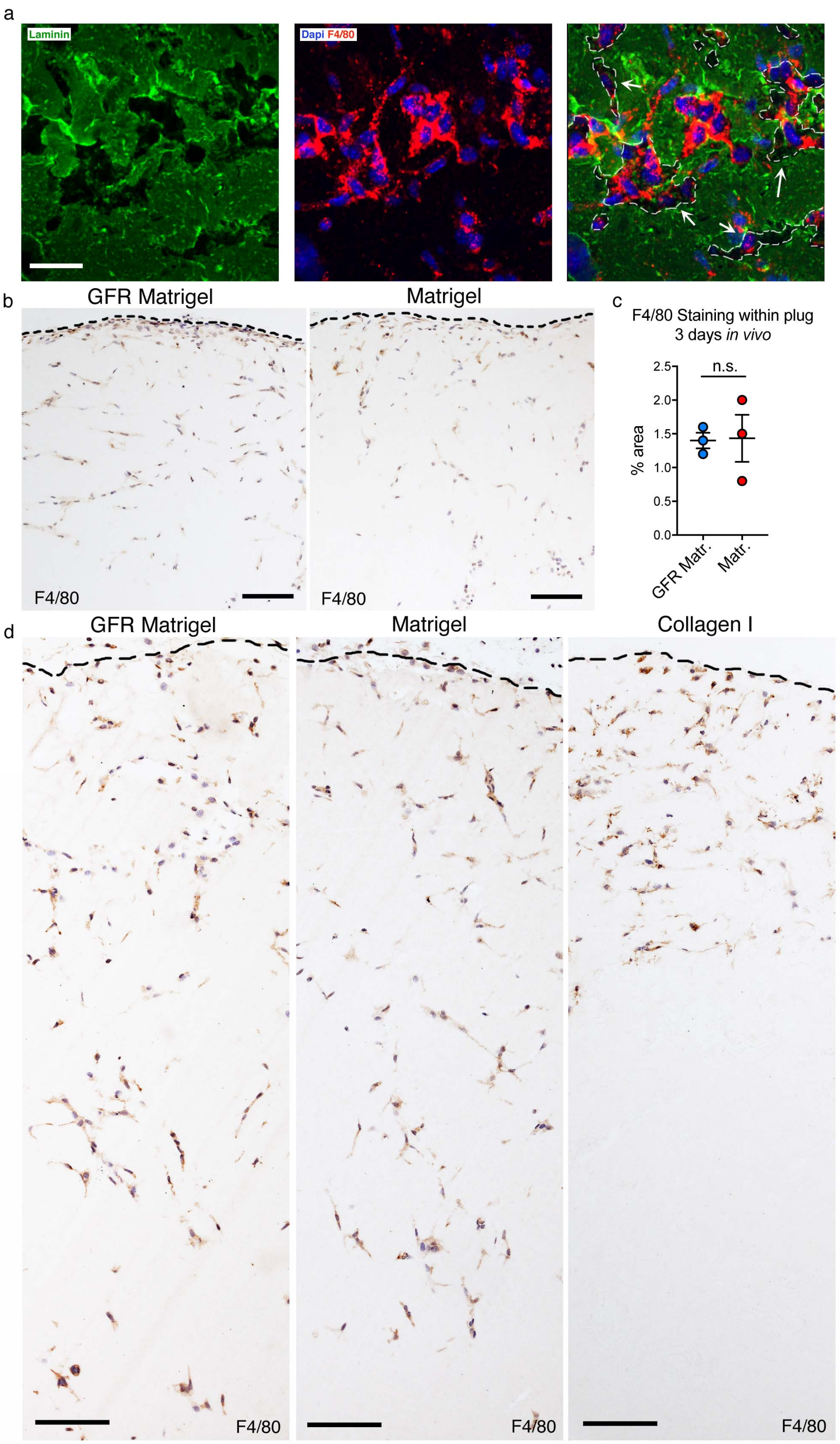
a
Laminin
Dapi F4/80
b
GFR Matrigel
Matrigel
F4/80
F4/80
c
F4/80 Staining within plug
3 days in vivo
n.s.
% area
2.5
2.0
1.5
1.0
0.5
0.0
GFR Matr.
Matr.
d
GFR Matrigel
Matrigel
Collagen I
F4/80
F4/80
F4/80

Supplementary Figure 4. Matrigel ECM components are the main trigger of macrophage recruitment.

a Representative immunofluorescence staining for laminin (green), F4/80 (red) and DAPI (blue) of PyMT plugs harvested 1 week post-transplantation. White lines and arrows show absence of Laminin. Scale bar: 25 μm. **b** Representative immunohistochemical staining for F4/80 3 days post-transplantation of either growth factor reduced (GFR) matrigel or standard matrigel. Scale bar: 100 μm. **c** Quantification of F4/80 staining of plug area in 4x images of either GFR or standard matrigel from b. n.s. P value>0.05 (n=3/group two tail T-test). **d** Representative immunohistochemical staining for F4/80 1 week post-transplantation of GFR matrigel, standard matrigel Collagen I plugs. Scale bar: 100 μm.

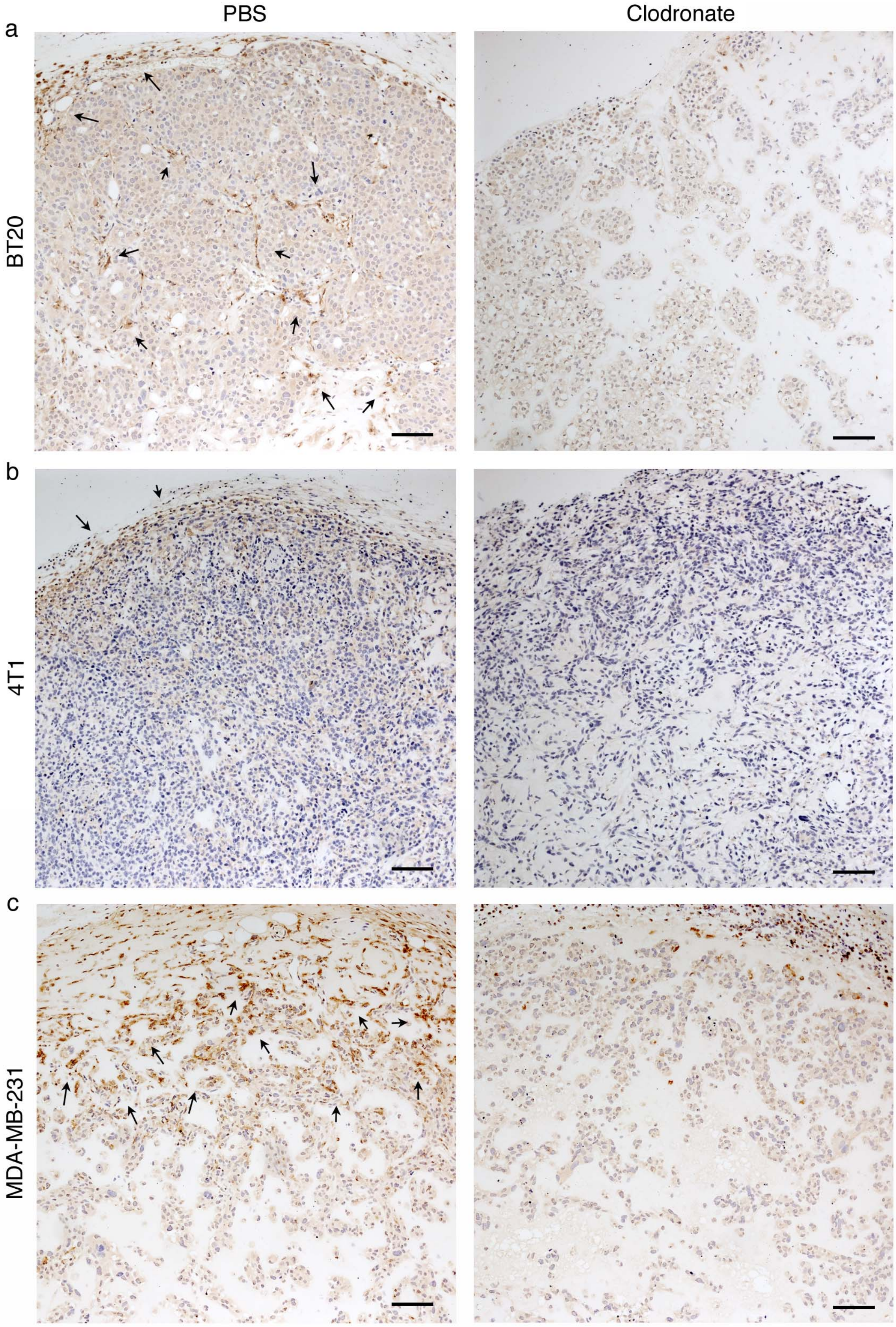

Supplementary Figure 5. Macrophage infiltration in plugs of human breast cancer lines.
Representative immunohistochemical staining for F4/80 of the different human breast cancer

lines growth either with PBS or Clodronate liposomes in Fig 6. **a** BT20 tumours; **b** 4T1 tumour; **c** MDAMB231 tumour. Scale bar: 100 μm. Arrows indicates F4/80 stained cells.
